# Principal component analysis of socioeconomic factors and their association with malaria in children from the Ashanti Region, Ghana

**DOI:** 10.1186/1475-2875-9-201

**Published:** 2010-07-13

**Authors:** Anne Caroline Krefis, Norbert Georg Schwarz, Bernard Nkrumah, Samuel Acquah, Wibke Loag, Nimako Sarpong, Yaw Adu-Sarkodie, Ulrich Ranft, Jürgen May

**Affiliations:** 1 Bernhard-Nocht-Institute for Tropical Medicine, Infectious Disease Epidemiology, Bernhard-Nocht-Straße 74, 20359 Hamburg, Germany; 2 Environmental Health Research Institute (IUF), Heinrich Heine University of Düsseldorf, Germany; 3 Kumasi Centre for Collaborative Research in Tropical Medicine, Kumasi, Ghana; 4 Kwame Nkrumah University of Science and Technology, School of Medical Sciences, Kumasi, Ghana

## Abstract

**Background:**

The socioeconomic and sociodemographic situation are important components for the design and assessment of malaria control measures. In malaria endemic areas, however, valid classification of socioeconomic factors is difficult due to the lack of standardized tax and income data. The objective of this study was to quantify household socioeconomic levels using principal component analyses (PCA) to a set of indicator variables and to use a classification scheme for the multivariate analysis of children < 15 years of age presented with and without malaria to an outpatient department of a rural hospital.

**Methods:**

In total, 1,496 children presenting to the hospital were examined for malaria parasites and interviewed with a standardized questionnaire. The information of eleven indicators of the family's housing situation was reduced by PCA to a socioeconomic score, which was then classified into three socioeconomic status (poor, average and rich). Their influence on the malaria occurrence was analysed together with malaria risk co-factors, such as sex, parent's educational and ethnic background, number of children living in a household, applied malaria protection measures, place of residence and age of the child and the mother.

**Results:**

The multivariate regression analysis demonstrated that the proportion of children with malaria decreased with increasing socioeconomic status as classified by PCA (p < 0.05). Other independent factors for malaria risk were the use of malaria protection measures (p < 0.05), the place of residence (p < 0.05), and the age of the child (p < 0.05).

**Conclusions:**

The socioeconomic situation is significantly associated with malaria even in holoendemic rural areas where economic differences are not much pronounced. Valid classification of the socioeconomic level is crucial to be considered as confounder in intervention trials and in the planning of malaria control measures.

## Background

Malaria is one of the major public health challenges subverting development in the poorest countries in the world. The direct and indirect costs of malaria are very high and the disease has played a significant role in the poor economic performance of sub-Saharan Africa. Sachs (2002) estimated, that the gross domestic product in these countries would be up to 32% greater today if malaria had been eliminated 35 years ago [1]. In contrast to a retrogressive trend of malaria morbidity and mortality in many areas malaria burden has been increasing in other areas [2]. Factors such as deteriorating health systems, growing drug and insecticide resistance, failure of water management but also socioeconomic, land-use factors, and climate are hypothesized to influence the emergence of malaria [3,4].

In Ghana, where the study was conducted, malaria is prevalent during the entire year and accounts for about 32-42% of all outpatient admissions and for major in-patient causes of death [5]. Sociodemographic factors such as ethnic group, parent's education and occupation, use of protective measures, and living standard of the family are suggested to be important risk factors for malaria and malaria epidemics [6-8]. The impact of socioeconomic factors, namely the family's financial situation, is difficult to assess due to the lack of standardized economic data of income and tax. The use of single indicators for the household's economical situation reduces the available information and may imperfectly set the focus point on the selected parameters. Additional socioeconomic factors assessed in the Demographic and Health Survey 2008 and not considered here are marital status and religion, which we did not found appropriate in the context of the study.

The aim of the presented study was to investigate the association between the socioeconomic status of families classified with a number of indicators as a PCA-based score and their association with childhood malaria.

## Methods

### Study area

**Figure 1:**
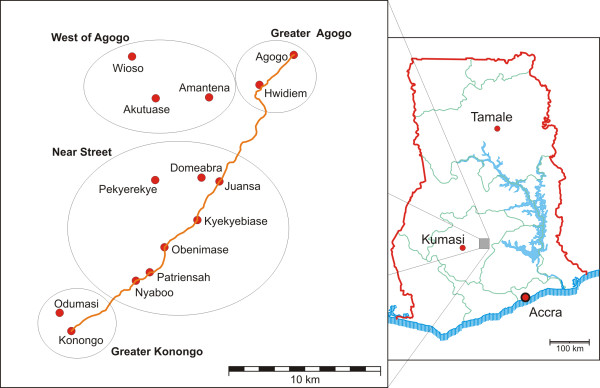
**Map of the 14 included villages and village clusters in the Asante Akim North District, Ashanti Region, central Ghana, West Africa**. Red dots indicate villages; the solid line indicates the main road.

This survey was accomplished at the Child Welfare Clinic and the Pediatric Ward of the Agogo Presbyterian Hospital, Asante Akim North District (Ashanti Region) in central Ghana, West Africa. Recruitment area included 14 villages which were summarized into 4 cluster ("Greater Agogo" (Agogo city and Hwidiem), "Greater Konongo" (Konongo and Odumasi), "West of Agogo" (Akutuase, Amantena and Wioso) and "Near Street" (Domeabra, Juansa, Kyekyebiase, Nyaboo, Obenimase, Patriensah and Pekyerekye) (Figure 1). The study area covers ~345 km^2^; the coverage population of the study hospital was 61,346 inhabitants (census data 2004) where the population ranged from 890 inhabitants in the smallest village to 15,383 in the largest.

The vegetation of the study area is mainly semi-deciduous forest with major vegetation types of open forest, closed forest and wooded savannah. The climate is tropical with a mean annual temperature of 26°C and two rainy seasons: a first rainy season from May to July and a second from September to November. The dry harmattan season occurs between December and April and is associated with drought conditions. The topography of the district is generally undulating and the altitude variation is 226 m between the lowest (227 m) and the highest (453 m) village included in our study. Agriculture is the predominant major occupation among people; main staple food crops produced in the district include maize, cassava, plantain, cocoyam and yam [9]. The principal malaria vectors are mosquitoes of the *Anopheles gambiae *complex and *Anopheles funestus*. Malaria is hyper-/holoendemic in this area with intense perennial transmission and seasonal peaks and the predominant *Plasmodium *species is *Plasmodium falciparum *(> 90%) [10]. Entomological evaluation during the study period indicated ~400 infective bites per person-year (EIR) (unpublished data). Subsidized insecticide-treated bed nets were available, and their use was encouraged.

The study was carried out between May 2007 and August 2009 (duration 26 months). Diagnostic assessments were integrated into the hospital routine. In total, 1,496 children up to 14 years of age, who visited the hospital for medical care, were included in the study. The case definition for malaria was fulfilled if the axillary temperature was ≥ 37.5°C and a *P. falciparum *parasitaemia count of > 0 parasites/μL was detected in the thick or thin smears. Parasite examination was done according to quality-controlled standardized procedures described elsewhere [11].

### Data collection

Information on personal or family characteristics with a possible influence on malaria (sex, ethnic group, age, mother's age, use of protective measures [usage of bed net, window net, other or no protection], number of children and place of residence) and information about factors indicating the family's financial situation (living in a brick or wood/mud house, existence of electricity, water supply, mother's education and profession, father's education and profession, indoor toilet and use of freezing as measure of conservation, income management, existence of a relative abroad for possible financial support and membership in the national Health Insurance Scheme [NHIS]) was collected through interviewing a parent or the guardian who accompanied the child to the hospital using a structured interview with a questionnaire in English or if necessary in the local language, Twi. The question sheet was composed according to standard questionnaires adjusted to local requirements and appropriateness. Data from questionnaires and forms were double entered after case closed, plausibility checked, and cleaned before the database was locked. All information on participants and their parents was treated confidentially. Only children who were examined for malaria and where information about the sociodemographic and socioeconomic situation was available were included in the analysis (n = 1496).

The study was approved by the Committee on Human Research, Publications, and Ethics, School of Medical Sciences, Kwame Nkrumah University of Science and Technology, Kumasi, Ghana.

### Data analysis

Participants were allocated into one of the four village clusters described above (Greater Agogo, Greater Konongo, West of Agogo and Near Street), according to their place of residence. Additionally, all participants were classified into two ethnic groups according to their tribal background: the Akan and those who are the natives of the area and the Northeners who have a migratory background but are now permanent residents of the area. It was hypothesized that children between the ages of 1 to 5 years are at highest risk of acquiring malaria; hence we stratified for age (≤ 1 year, > 1 to ≤ 5 years and > 5 years). It was also suggested that the mother's age might be of importance for the child and its risk for malaria; all mothers were stratified and grouped to young mothers (≤ 30 years) and older mothers (> 30 years). High numbers of children living in a household were assumed as an influence factor (two groups: ≤ 4 children and > 4 children). Additionally, it was asked in the interview whether a family used protective measures such as bed nets or window-screens. Individuals with missing values on any of these variables (n = 18) were excluded from the analysis (n = 1478).

To classify the family's economic status, the following socioeconomic indicator variables were considered: mother's and father's profession (employed/unemployed) and education (ability to read and write: yes/no), type of house the family is living in (cement/brick house or mud/wood house), water supply (open water source/closed water source), existence of an indoor kitchen (Yes/No), electricity (Yes/No), indoor toilet (Yes/No), use of freezing as measure of conservation (Yes/No), existence of a relative abroad who might financially support the family (Yes/No), the self-rated ability to manage with the available monthly income (difficult or not difficult) as well as the membership in the health insurance (Yes/No). All socioeconomic and sociodemographic data including information on protective measures based on self-reports of the mothers or guardians and were not confirmed by direct observations during household visits.

For the sake of the multivariable analysis, a principal component analysis (PCA) was applied to those socioeconomic indicator variables, which showed relevant contributions (> 10%) to the combined socioeconomic status score factor [12]. The factor of the PCA with the highest eigenvalue was used as the variable, which describes sufficiently the socioeconomic status of a household. The respective factor scores were categorized in terciles and used in the regression analysis. The lowest 33% of households according to the economic status variable were classified as poor, the highest 33% as rich and the rest as average economic status [13].

For the PCA, missing values of distinct binary variables were replaced by the means of all summarized "0" values (asset not present) and "1" values (asset present) of this variable (n = 1496) [12]. This approach may have reduced variation among households and may have increased the potential for clumping and truncation [12,14]. In the presented study population, the percentage of households with missing values was, however, small (< 1%) and such a bias might be negligible.

For each potential risk factor of malaria, the odds ratio (OR) was calculated and the significance level was tested by the chi-square test. Adjusted ORs were estimated by multivariate logistic regression. Confounding was determined as a relative difference of 15% between crude odds ratios and odds ratios adjusted for predefined covariates without signs of effect modification. All covariables in the multivariate regression model were examined for possible effect modification by Wald tests and preference of the model with interaction by log-likelihood tests (both p < 0.05).

## Results

In total, 1,496 children were examined for malaria parasites and participated in the questionnaire survey; 1,478 without missing values were included in the multivariate model. Most participants came from the region Greater Agogo (n = 871), fewer came from Greater Konongo (n = 333), Near Street (n = 229), and West of Agogo (n = 63). Most malaria cases were reported from Greater Agogo (n = 364), followed by Greater Konongo (n = 66), Near Street (n = 61), and West of Agogo (n = 21) (See additional file 1 describing characteristics of the study group).

Of those variables with a possible influence on malaria use of individual control measures had a protective effect on malaria (crude OR = 0.69, p = 0.02) (See additional file 1 describing characteristics of the study group). Malaria odds were increased if a child was between > 1-< 5 or above 5 years of age (OR = 3.41, and OR = 2.07, both p < 0.001). Notably, the area of residence was strongly associated with the frequency of malaria (OR Greater Agogo compared to Greater Konongo = 3.1, p < 0.001). The variables "ethnic groups", "sex", "mother's age" and "number of children in the family" did not show any significant association with malaria.

Of those factors indicating the family's socioeconomic status the proportion of literate fathers was very high (> 75%) and evenly distributed under children with and without malaria. Likewise, the variables "house type", "income manage", "membership in a health insurance", "existence of an indoor kitchen" and "mother's and father's occupation" did not show any distinct association with malaria (See additional file 1 describing characteristics of the study group). In the univariate analysis, the variables "existence of electricity", "indoor toilet", "use of freezing as food conservation", "mother's ability to read and write" and a "closed water supply" were negatively associated with malaria odds (OR = 0.72, OR = 0.67, OR = 0.63, OR = 0.68, OR = 0.70, respectively, all p-values < 0.01). Additionally, the variable "existence of a relative abroad" known to be indicative for a substantive contribution to the household income in Africa had a protective effect on malaria (p = 0.05). All variables with relevant contributions (> 10%) to the combined socioeconomic score were used to generate a combined socioeconomic indicator by PCA; hence "mother's occupation" and "father's occupation" were excluded from the final PCA (weight mother's occupation and father's occupation: 5% and 4%, respectively).

The results of the PCA are presented in Table 1. The eigenvalues demonstrated that one principal factor had a weight greater than two (2.20) and thus was suited to appropriately represent the socioeconomic status in further analyses. This variable (factor 1 in Table 1), now interpreted as a socioeconomic score, explained 20% of the variance of the eleven original variables. All variables included in the PCA had positive factor scores, and therefore were associated with higher socioeconomic status. Freezing as measure of conservation had, with a weight of 0.65, the highest contribution to the combined socioeconomic status score (Table 1), membership in the NHIS had the lowest impact on the combined indicator with a weight of 0.15. For further analyses, we classified the socioeconomic score in three categories using tertiles: "poor", "average" and "rich". The village cluster Greater Agogo and Near Street had the highest proportion of households considered poor with 38% and 35%, respectively. In Greater Konongo was the highest proportion of households categorized as being rich (47%). Most malaria cases were reported from individuals classified as "poor" (n = 202, 39%) followed by those grouped as "average" (n = 189, 37%) and "rich" (n = 121, 24%).

**Table 1:** Results from the principal component analysis (PCA)

Factor	Eigenvalue	Variance proportion	Cumulative variance proportion
Factor 1	2.20	0.20	0.20
Factor 2	1.07	0.10	0.30
Factor 3	1.06	0.10	0.40
Factor 4	1.01	0.09	0.49
Factor 5	0.97	0.09	0.57
Factor 6	0.94	0.09	0.66
Factor 7	0.85	0.08	0.74
Factor 8	0.82	0.07	0.81
Factor 9	0.76	0.07	0.88
Factor 10	0.71	0.06	0.94
Factor 11	0.62	0.06	1.00

**Observed variable**	**Weight for factor 1 (economic status score)**		

Freezing as conservation	0.65		
Education mother	0.58		
Toilet supply	0.56		
Electricity	0.53		
House type	0.47		
Education father	0.43		
Income manage	0.43		
Relative abroad	0.33		
Water supply	0.27		
Cooking	0.22		
NHIS	0.15		

Additionally, clumping and truncation (if the distribution of scores tend not to follow a normal curve or if they were skewed) was checked by using a histogram to show the distribution of socioeconomic scores. Internal coherence for our study region could be shown, suggesting appropriate and sufficient choice of asset variables.

All potential risk factors, together with the newly created variable describing the socioeconomic status were included in the final logistic regression model to assess their independent effect on malaria risk (Table 2). In the full multivariable model, an independent association was seen for the family's socioeconomic status. In comparison to the poor group, belonging to the group of average socioeconomic status decreased the odds to 0.88 (p = 0.35), and being rich decreased the odds for malaria further to 0.56 (p < 0.001). The results remained consistent in the parsimonious stepwise logistic regression. All significant risk factors were checked for effect modification, but none could be detected.

**Table 2:** Influence of socioeconomic and sociodemographic factors on malaria in a multivariate logistic regression analysis.

			Stepwise logistic Regression			
Determinants	**OR**^1^	CI	p-value	**OR**^1^	CI	p-value
Reference*	1					
Economic status^2^						
'average'	0.88	0.66 - 1.16	0.35	0.88	0.67-1.15	0.34
'rich'	0.56	0.41 - 0.76	< 0.001	0.56	0.42-0.75	< 0.001
Use of protection measures^3^	0.71	0.51 - 1.00	0.05	0.72	0.51-1.00	0.05
Age < 1 - ≤ 5 years	3.34	2.57-4.36	< 0.001	3.39	2.61-4.40	< 0.001
Age > 5 years	2.10	1.52 - 2.88	< 0.001	2.04	1.51-2.75	< 0.001
Place of Residence						
West of Agogo	0.77	0.43 - 1.37	0.38	0.78	0.44-1.37	0.38
Near street	0.52	0.37 - 0.74	< 0.001	0.51	0.36-0.72	< 0.001
Greater Konongo	0.39	0.28- 0.53	< 0.001	0.39	0.29-0.54	< 0.001
Ethnic group	0.90	0.63 - 1.29	0.58			
Number of children	1.19	0.87- 1.64	0.28			
Sex	0.88	0.70 - 1.10	0.26			
Mother's age	1.02	0.78-1.33	0.91			

*Reference: Economic status ‚poor', no use of protection measures, age ≤ 1 year, place of residence ‚Greater Agogo', Ethnic group ‚Northeners, > 4 children, sex: male, mother age ≤ 30 years of age.

^1^Odds ratio mutually adjusted with all other variables in a multivariable logistic regression

^2^Economic status classified by using factor 1 of PCA (Table 1)

^3^Reported protection measures such as bed net or window fences

## Discussion

The analysis showed that, in an area of high endemicity, the proportion of malaria in children presented to a hospital is markedly influenced by the socioeconomic status of the family: children from households classified as poor had a significantly higher chance to get malaria. This is in agreement with previous reports on distinct socioeconomic risk factors for malaria [6-8,15,16]. One possible explanation of this observation is that the proportion of children using protective bed nets increases with the socioeconomic status as reported before [17,18]. However, after adjustment for the use of bed nets in the multivariate analysis an association of the socioeconomic status with malaria still remained. Other possible explanations for the association between malaria and socioeconomic status are (i) differences in the coverage of health insurance [19] which was, however, not of significant influence in the univariate analysis, (ii) differing access to health facilities, whereas such a selection bias might be low due to the hospital-based study design, (iii) various environmental or housing conditions in the vicinity of households e.g. preferred habitats or breeding sites for vectors what is difficult to exclude [20,21].

Socioeconomic levels might also be associated with diseases beyond malaria and may influence the proportion of malaria cases among all children seen in the hospital. This would have an indirect influence on the calculated odds ratios. However, the two symptom complexes predominant in children without malaria, namely respiratory distress and gastrointestinal symptoms, were not or only weakly associated with socioeconomic levels. The study design, which bases on a single hospital, might limit the generalization of the results to other regions. On the other hand, the focus on one hospital allowed the thorough collection of data on the clinical condition, infectious disease agents, and exact diagnosis.

One problem of the determination of individual socioeconomic levels in Africa is the fact that unambiguous quantitative measures often do not exist and various proxy measures must be used as an approximation. The use of single variables as risk indicators often leads to false conclusions because they only reflect parts of the general view. In contrast, in PCAs socioeconomic indicator variables were combined to enable a quantification and classification of individual socioeconomic levels and to use the resulting score for risk analyses. The PCA showed that usage of a freezer as conservation method, which was interpreted as ownership of a freezer in a household, had the highest weight for the socioeconomic score. On the other hand, having a health insurance was the smallest compared to the other ten variables and, hence, was of a minor importance for the socioeconomic score.

An advantage of the PCA is that it reduces measurement problems, such as recall bias, and that it reduces the complexity of correlated data, which can be easily collected as single indicator variables in household surveys [12,22]. On the other hand, the process of generalization leads to a loss of information, the criteria for the selection of variables for PCA are not well defined, and the number of selected components is arbitrary. Whether a single principal component can sufficiently determine the socioeconomic status is entirely dependent on the data and the correlation matrix of the variables, their validity and reliability [12].

Apart from the socioeconomic status, sociodemographic factors were associated with malaria. As expected the malaria risk was highest in the age group of children between 1 and 5 years, compared to children below the age of 1 year [23] and lower in children from families, which reported the use of mosquito protection measures [16,24]. There was a decrease of odds for malaria with increasing distance from the study hospital. A simple selection bias is not a sufficient explanation for that observation since all children included had access to the hospital. However, it is conceivable that the willingness to bring a child with malaria symptoms decreases with increasing distance from the study hospital and, in contrast, the readiness to bring a child with other symptoms is more independent from distance. If so, the relative contribution of malaria cases decreases with distance and this would falsely suggest a protective effect of distance against malaria. Nevertheless, data from a recently performed community survey in the same area showed that the health seeking behaviour among differing symptoms did not change with distance. It was not possible to include the village population size as influence factor due to the village-cluster based analysis. Geographical risk factors seem to exist independently from other influences, maybe through environmental and habitat factors favouring the occurrence of vectors. To assess environmental influence factors remotely sensed data with high resolution should be analysed with Geographical Information System (GIS) to detect microspatial patterns in relation to malaria risk.

Age of mothers did not influence the occurrence of malaria of their children. However, in the study group only 3% of the mothers were younger than 20 years of age, which does not represent the mother's age distribution in Ghana or other African countries. Although ethnicity was found to influence malaria risk in a study conducted in an adjacent region in Ghana [6] this could not be confirmed in the presented study possibly due to the predominance of one ethnic group.

## Conclusions

In conclusion, the herein presented results show that children from poorer households are of greater risk for malaria. It is under discussion how far poverty influences the occurrence of malaria or malaria influences the occurrence of poverty. In either case, the fight against malaria has to be escorted by the fight against poverty and improvement of living standard. Moreover, the spatial variability of malaria risk might be of importance for the planning of control measures and the conduction of intervention trials.

## Competing interests

The authors declare that they have no competing interests.

## Authors' contributions

ACK participated in the conception, performed the statistical analysis, was involved in the interpretation of results and participated in drafting the results. NGS participated in the design, assisted in performing the statistical analysis, was involved in the interpretation of the results, and helped to draft the manuscript. WL created Case Report Forms and was responsible for data management and data preparation for analyses. UR was involved in the initial design of the study, assisted in performing the statistical analysis and participated in drafting the manuscript. JM conceived and coordinated the study, was involved in the initial design of the study and writing of the manuscript. NS organized the day-to-day work on the ground, BN and SA carried out the malaria parasite examination and contributed to the writing of the manuscript, and YAS planned, initiated the study, and reviewed manuscript. All authors have read and approved the final manuscript.

## Supplementary Material

Additional file 1 **Characteristics of the study group**. the table shows the results of a first univariate analysis including variables which give information on personal or family characteristics with a possible influence on malaria and information about factors indicating the family's financial situation. Click here for file
