# Supplementary material for: Principal component analysis of socioeconomic factors and their association with malaria in children from the Ashanti Region, Ghana
Source: Malar J. 2010 Jul 13;9:201. doi: 10.1186/1475-2875-9-201 (PMC2914064; doi:10.1186/1475-2875-9-201)
Supplement: Additional file 1 — Characteristics of the study group. the table shows the results of a first univariate analysis including variables which give information on personal or family characteristics with a possible influence on malaria and information about factors indicating the family's financial situation. [file 1475-2875-9-201-S1.DOC]

**Table 1** Characteristics of the study group

| Characteristic | Malaria yes | Malaria no | OR | CI | p-value |
| --- | --- | --- | --- | --- | --- |
|  | n=512 | n=984 |  |  |  |
| Sex |  |  |  |  |  |
| Male | 278 (35%) | 522 (65%) | 1 |  |  |
| Female | 234 (34%) | 462 (66%) | 0.95 | 0.77 – 1.18 | 0.65 |
| Place of residence |  |  |  |  |  |
| Greater Agogo | 364 (42%) | 507 (58%) | 1 |  |  |
| West of Agogo | 21 (33%) | 42 (67%) | 0.70 | 0.41 – 1.20 | 0.19 |
| Near Street | 61 (27%) | 168 (73%) | 0.51 | 0.37 – 0.70 | <0.001 |
| Greater Konongo | 66 (20%) | 267 (80%) | 0.34 | 0.25 – 0.47 | <0.001 |
| Ethnic group^1^ |  |  |  |  |  |
| Northeners | 70 (39%) | 108 (61%) | 1 |  |  |
| Akan and others | 441 (33%) | 876 (67%) | 0.78 | 0.56 – 1.07 | 0.12 |
| Age child |  |  |  |  |  |
| ≤ 1 year of age | 147 (22%) | 522 (78%) | 1 |  |  |
| >1-≤ 5 years of age | 244 (49%) | 254 (51%) | 3,41 | 2.65 - 4.40 | <0.001 |
| > 5 years of age | 121 (37%) | 208 (63%) | 2.07 | 1.55 – 2.76 | <0.001 |
| Age mother |  |  |  |  |  |
| ≤ 30 years of age | 265 (33%) | 540 (67%) | 1 |  |  |
| > 30 years of age | 247 (36%) | 444 (64%) | 1.13 | 0.92 – 1.40 | 0.25 |
| Protective measure |  |  |  |  |  |
| No protection | 73 (42%) | 101 (58%) | 1 |  |  |
| Protection^2^ | 437 (33%) | 878 (67%) | 0.69 | 0.50 – 0.95 | 0.02 |
| Mother´s occupation |  |  |  |  |  |
| Unemployed | 63 (37%) | 107 (63%) | 1 |  |  |
| Employed | 442 (34%) | 867 (66%) | 0.87 | 0.62 – 1.21 | 0.39 |
| Father´s occupation |  |  |  |  |  |
| Unemployed | 5 (28%) | 13 (72%) | 1 |  |  |
| Employed | 498 (34%) | 966 (66%) | 1.34 | 0.47 – 3.78 | 0.58 |
| Mother´s education^3,5^ |  |  |  |  |  |
| No | 340 (37%) | 571 (63%) | 1 |  |  |
| Yes | 165 (29%) | 410 (71%) | 0.68 | 0.54 – 0.85 | <0.001 |
| Father´s education^3,5^ |  |  |  |  |  |
| No | 123 (38%) | 200 (62%) | 1 |  |  |
| Yes | 375 (33%) | 774 (67%) | 0.79 | 0.61 – 1.02 | 0.07 |
| Water supply^4,5^ |  |  |  |  |  |
| Open water | 118 (41%) | 170 (59%) | 1 |  |  |
| Closed water | 392 (33%) | 812 (67%) | 0.70 | 0.53 – 0.91 | 0.007 |
| Children, no |  |  |  |  |  |
| > 4 children | 103 (35%) | 188 (65%) | 1 |  |  |
| ≤ than 4 children | 403 (34%) | 792 (66%) | 0.93 | 0.71 – 1.21 | 0.59 |
|  |  |  |  |  |  |
|  | | | | | |
| House type^5^ |  |  |  |  |  |
| Mud/wood | 61 (31%) | 137 (69%) | 1 |  |  |
| Brick/cement | 449 (35%) | 846 (65%) | 1.19 | 0.86 – 1.65 | 0.29 |
| Income manage^5^ |  |  |  |  |  |
| Difficult | 295 (36%) | 526 (64%) | 1 |  |  |
| Not difficult | 210 (32%) | 455 (68%) | 0.82 | 0.66 – 1.02 | 0.08 |
| Health insurance^5^ |  |  |  |  |  |
| No | 126 (32%) | 265 (68%) | 1 |  |  |
| Yes | 365 (35%) | 682 (65%) | 1.13 | 0.88 – 1.44 | 0.35 |
| Cooking^5^ |  |  |  |  |  |
| Outside | 143 (33%) | 288 (67%) | 1 |  |  |
| Inside | 367 (35%) | 695 (65%) | 1.06 | 0.84 – 1.35 | 0.61 |
| Electricity^5^ |  |  |  |  |  |
| No | 139 (40%) | 209 (60%) | 1 |  |  |
| Yes | 367 (32%) | 768 (68%) | 0.72 | 0.56 – 0.92 | 0.009 |
| Toilet supply^5^ |  |  |  |  |  |
| Outdoor | 264 (39%) | 409 (61%) | 1 |  |  |
| Indoor | 248 (30%) | 575 (70%) | 0.67 | 0.54 – 0.83 | <0.001 |
| Relative abroad^5^ |  |  |  |  |  |
| No | 419 (35%) | 764 (65%) | 1 |  |  |
| Yes | 89 (29%) | 214 (71%) | 0.76 | 0.58 – 1.00 | 0.05 |
| Freezer usage^5^ |  |  |  |  |  |
| No | 379 (37%) | 639 (63%) | 1 |  |  |
| Yes | 116 (27%) | 312 (73%) | 0.63 | 0.49 – 0.80 | <0.001 |
| Economic status^6^ |  |  |  |  |  |
| poor | 202 (41%) | 287 (59%) | 1 |  |  |
| average | 189 (36%) | 332 (64%) | 0.81 | 0.63 – 1.04 | 0.101 |
| rich | 121 (25%) | 365 (75%) | 0.47 | 0.36 – 0.62 | 0.000 |

OR: odds ratio, CI: Confidence Interval, p-value: chi-square test

^1^ Ethnic groups „Others“: Ewe, Ga, Other, not clear

^2^ Protection: Bed net, Other, Window net

^3^ Education: Ability to read and write

^4^ Closed water source = Inside tap or Stand pipe; open water source = River or well

^5^ Variables included in the Principal Component Analysis

^6^ Computed by means of Principal Component Analysis
